# Development of a robust MRI fiducial system for automated fusion of MR‐US abdominal images

**DOI:** 10.1002/acm2.12352

**Published:** 2018-05-21

**Authors:** Christopher P. Favazza, Krzysztof R. Gorny, Matthew R. Callstrom, Anil N. Kurup, Michael Washburn, Pamela S. Trester, Charles L. Fowler, Nicholas J. Hangiandreou

**Affiliations:** ^1^ Department of Radiology Mayo Clinic Rochester MN USA; ^2^ GE Healthcare Wauwatosa WI USA

**Keywords:** auto‐registration, fiducial marker, image fusion, MRI, ultrasound

## Abstract

We present the development of a two‐component magnetic resonance (MR) fiducial system, that is, a fiducial marker device combined with an auto‐segmentation algorithm, designed to be paired with existing ultrasound probe tracking and image fusion technology to automatically fuse MR and ultrasound (US) images. The fiducial device consisted of four ~6.4 mL cylindrical wells filled with 1 g/L copper sulfate solution. The algorithm was designed to automatically segment the device in clinical abdominal MR images. The algorithm's detection rate and repeatability were investigated through a phantom study and in human volunteers. The detection rate was 100% in all phantom and human images. The center‐of‐mass of the fiducial device was robustly identified with maximum variations of 2.9 mm in position and 0.9° in angular orientation. In volunteer images, average differences between algorithm‐measured inter‐marker spacings and actual separation distances were 0.53 ± 0.36 mm. “Proof‐of‐concept” automatic MR‐US fusions were conducted with sets of images from both a phantom and volunteer using a commercial prototype system, which was built based on the above findings. Image fusion accuracy was measured to be within 5 mm for breath‐hold scanning. These results demonstrate the capability of this approach to automatically fuse US and MR images acquired across a wide range of clinical abdominal pulse sequences.

## 1 INTRODUCTION

Magnetic resonance imaging (MRI) provides outstanding contrast resolution of most visceral soft‐tissue tumors, and consequently, it is very commonly used in the abdomen or pelvis for lesion detection and characterization. However, application of MRI for guidance of percutaneous diagnostic or interventional procedures such as biopsy or thermal ablation remains a challenge due to limited access within the MRI scanner bore and incompatibility of some percutaneous devices with the MRI environment. Instead, ultrasound (US) is more commonly used as a guidance modality because it is widely accessible and portable for use in procedural suites. Moreover, US guidance affords flexibility in angle of approach and allows precise, real‐time targeting of lesions in spite of normal respiratory motion. Unfortunately, lesions may have low conspicuity with US imaging, particularly in obese patients, in patients with prior chemotherapy treatment or in patients with diffuse parenchymal disease.^1^, ^2^, ^3^ Thus, fusion or co‐registration of real‐time US with previously obtained MRI has been advocated for guidance of percutaneous interventions on challenging lesions. MR‐US image fusion pairs the advantages of MRI, namely high‐contrast resolution and lesion conspicuity, with the real‐time capabilities of US guidance, and has been shown to be clinically beneficial.^1^, ^2^, ^3^, ^4^, ^5^, ^6^, ^7^, ^8^

Most current commercial MR‐US image fusion applications rely on manual co‐registration of images. (One example is General Electric's LOGIQ E9 ultrasound system with Volume Navigation^9^) For instance, anatomical structures that are clearly discernible in the MR image and a real‐time US image are manually identified for registration of the two image sets. An electromagnetic sensor tracks the position and orientation of the US probe, and these data are used to create the appropriate multiplanar reconstructed image from the MR image set for fusion with the real‐time B‐mode US image.

Limitations of current MR‐US fusion systems include difficulty accurately identifying common landmarks in the MR and US imaging, time necessary for point‐to‐point registration, and registrations that are inaccurate in all three planes. An automated system could expedite MR‐US image registration and reduce the error of the registered imaging. Several automated MR‐US fusion approaches, based on external MR fiducial markers, have been investigated. With these approaches, accurate image fusion hinges upon reliable segmentation of an MR fiducial device. External fiducial devices comprised a known arrangement of active or passive signal‐generating markers have been shown to facilitate an automated registration process.^10^, ^11^, ^12^, ^13^ Active fiducial marker devices are limited in that they are complex, are susceptible to radio‐frequency heating during MRI, and rely on precise tuning and calibration.^10^ On the other hand, recently investigated passive marker systems^10^, ^11^, ^12^, ^13^ required use of custom or specific MRI pulse sequences with some relying on spatial frequency images^10^, ^11^ or only being limited to specific clinical application (i.e. fixed in headrest for intracranial imaging only).^13^ Additional approaches to MR‐US fusion without the use of external fiducial markers have also been reported.^14^, ^15^ These methods, however, require either acquisitions of 3D US images combined with extensive computation time and initial manual three‐point rigid registration,^14^ or have been demonstrated to be successful only with a single MRI pulse sequence.^14^, ^15^

In a proposed system, automated MR‐US image fusion works as follows: an MRI fiducial device attached to a patient is imaged in an MR scanner, and subsequently, automatically segmented within the acquired image set. When the patient undergoes a US‐guided interventional procedure, an electromagnetic sensor is attached to the fiducial and placed on the patient in the same location as it was during the prior MRI examination. The position of the US transducer is then known relative to the fixed fiducial and, consequently, known relative to the MR image set (assuming the MRI fiducial device is accurately located). The real‐time US images are then directly fused with the MRI images without the need for manual identification of shared anatomical landmarks. This image fusion concept has been successfully developed for CT‐US image fusion applications and is commercially available.^1^

In this article, we present development of a MRI fiducial system, comprising a passive MRI fiducial marker device and a corresponding autosegmentation algorithm. The individual fiducial markers were designed to yield high signal‐to‐noise ratio (SNR) values across a wide range of MR pulse sequences, patient sizes, and acquisition geometries. Likewise, the segmentation algorithm was designed to be robust and function well for different image acquisition parameters. The initial application of this work is to couple the fiducial device‐algorithm system with a commercial US scanner capable of manual MR‐US image fusion, such as General Electric's LOGIQ E9 ultrasound and Volume Navigation systems,^9^ allowing automated MR‐US image fusion to be achieved. However, the fiducial device and auto‐segmentation algorithm should be generally applicable to other clinical problems in which automatic registration of MR data sets is beneficial.

## 2 MATERIALS AND METHODS

### 2.A Fiducial device prototype

**Figure 1:**
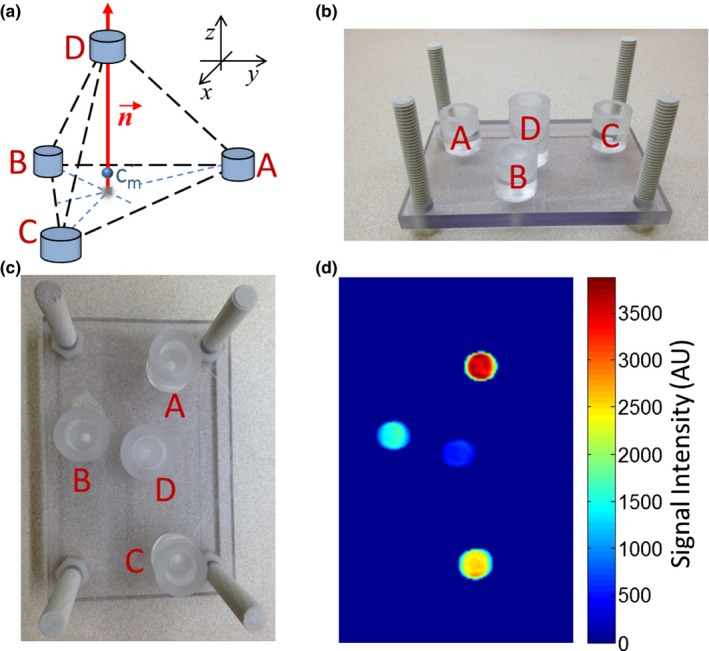
(a) Schematic of fiducial device prototype, used in the experiments described in Section 2.C of the Methods. The red vector, $\vec{n}$, indicates the orientation of the device, that is, the vector normal to the base formed by three co‐planar markers. The point, c_m_, indicates the center‐of‐mass of the device. (b) Photo of the fiducial device prototype from a side view [Different scale than in (a)]. (c) Photo of the fiducial device prototype. Letters serve to identify each marker. (d) Corresponding sample maximum intensity projection image of the segmented fiducial device acquired with the SS‐FSE pulse sequence.

The fiducial marker device was designed with the goal of being detectable in images acquired with the wide array of possible pulse sequences used in our institution's clinical abdominal MRI protocol (Table 1). The prototype device, shown in Fig. 1, consisted of three cylindrical reservoirs with 12.7 mm inner diameter and depth, arranged to form a scalene triangle with the following side lengths: 50.7, 69.2, and 88.9 mm. A fourth reservoir was positioned 12.7 mm above the centroid of the triangle as shown in Fig. 1(a). Actual marker separation distances were within an estimated tolerance of 1 mm. The reservoirs were filled with 1 g/L (6.265 mM) copper sulfate solution. The location of the device was defined as the center‐of‐mass of the group of four markers (derived from their individual, intensity‐weighted, center‐of‐mass coordinates), shown in Fig. 1(a). The orientation of the device was defined as the cross product of the vectors from marker A to marker B and marker A to marker C, see Fig. 1(c).

**Table 1:** Select parameters of the pulse sequences investigated

MRI Parameter	3 plane loc.	SS‐FSE	IP/OP	LAVA	FIESTA	SPGR	FR‐FSE
TE (ms)	79.7	86.1	2.2; 4.4	1.8	1.6	3.6	117.2
TR (ms)	880.2	1153.1	140.0	3.9	4.5	135.0	1916.7
Flip Angle (°)	90.0	90.0	70.0	15.0	75.0	70.0	90.0
X‐res. (mm)	1.9	0.9	0.7	0.7	0.7	0.7	0.7
Y‐res. (mm)	1.9	0.9	0.7	0.7	0.7	0.7	0.7
Z‐res. (mm)	10.0	6.0	6.0	1.7	4.0	5.0	7.0
Slice gap (mm)	0	1	1	NA	2	1	1
Scan plane	Ax; Cor; Sag	Cor	Ax	Ax	Ax	Ax	Ax
Acquisition	2D	2D	2D	3D	2D	2D	2D

**Figure 2:**
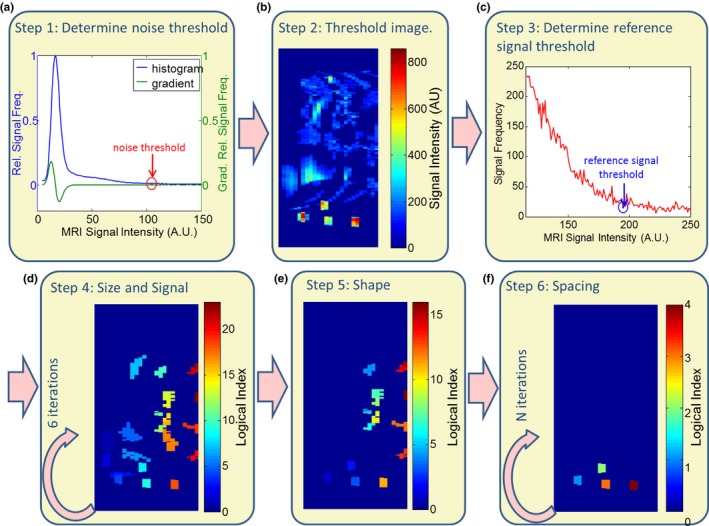
Flow chart describing the primary steps of the automated detection algorithm.

The choice of 1 g/L copper sulfate solution as a fiducial marker material was motivated by published reports indicating this material as suitable for preparation of high‐contrast MRI markers.^16^, ^17^ In addition, liquid copper sulfate solutions are readily available and convenient for fabricating custom fiducial markers. The particular selection of the marker size of 12.7 mm took into account the average slice thickness and slice gap of the RF pulse sequences listed in Table 1: 5–6 mm and 1–2 mm, respectively (Fig. 2).

### 2.B Segmentation algorithm

Operational steps of the MATLAB^®^ (The MathWorks Inc., Natick, MA, 2000) algorithm used to automatically segment markers from MRI datasets are described below and in Fig. 2. The empirically determined parameters associated with each step are listed in Table 2.

**Table 2:** Segmentation algorithm parameters

Algorithm step	Input	Discriminator		Returned value
		Type	Value	
1. Determine “noise threshold”	Raw 3D image	Voxel intensity histogram and its derivative	1st zero‐crossing—after first peak histogram value	Noise threshold. 3D image containing only voxels with intensities above noise threshold
2. Segment “connected voxel objects”	Raw 3D image	Size of connected‐voxel objects (*V*_0_)—26 voxel neighborhood	$\frac{1}{2}V_{\mathrm{marker}}<V_{0}<3V_{\mathrm{marker}}$	Size thresholded 3D image
3. Determine “reference threshold”	Background‐Size thresholded 3D image	–	–	Reference threshold (*R*_*T*_)—average intensity value of marker‐sized objects
4. Size and Signal discrimination (6 iterations)	(a) Background‐thresholded 3D image	Voxel intensity value	$S_{T}^{n}=\frac{n}{4}\times R_{T};n=7,6,5,4,3,2$	Signal‐thresholded 3D image
	(b) Signal‐thresholded 3D image	Size of connected voxel objects—26 voxel neighborhood	$0.35V_{\mathrm{marker}}<V_{0}<1.3V_{\mathrm{marker}}$	Signal‐Size thresholded 3D image
	(c) For 2nd–6th iterations: Signal‐Size + Composite Signal‐Size thresholded 3D images	Size of connected voxel objects—26 voxel neighborhood	$0.35V_{\mathrm{marker}}<V_{0}<1.3V_{\mathrm{marker}}$	Composite Signal‐Size thresholded 3D image
5. Shape discrimination	Composite Signal‐Size thresholded 3D image	Candidate marker linear dimensions (x, y, and z logical axes)	<1.5 × longest marker dimension	Shape thresholded 3D image
6. Inter‐Marker Distance discrimination (N iterations)	Shape thresholded 3D image	Distance between centers‐of‐mass of candidate marker and all other candidate markers	True marker separation ± n (mm) marker separation distances	(a) 3D image of the 4‐marker fiducial device (b) Fiducial device center‐of‐mass and orientation

#### 2.B.1 Step 1: Determination of “noise threshold,” $N_{T}$

The purpose of this step is to remove the majority of voxels containing background noise only. A histogram of voxel intensity values (bin size set to single integer and number bins set to the maximum voxel signal), $h\left(I\right)$, is expected to contain a major peak at a low‐intensity value, *I*_*noise*_, which is associated with image noise. Additional peaks at higher intensity values, *I*_*signal*_, are associated with MRI signal. As a result, the derivative of the histogram will change sign at some intermediate intensity value, *I*_0_, between the low‐intensity and high‐intensity peaks. We define the noise threshold as the voxel value corresponding to this zero‐crossing of the histogram derivative: *N*_*T*_ = *I*_0_ for *I*_0_ such that *I*_*noise*_ < *I*_0_ < *I*_*signal*_;$h^{\prime }\left(I_{0}\right)=0$; $h^{\prime }\left(I\right)<0$ for *I* < *I*_0_ and $h^{\prime }\left(I\right)>0$ for *I* > *I*_0_. All voxels with values below the noise threshold are set to zero in the image.

#### 2.B.2 Step 2: Determination of reference signal threshold, *R*_*T*_

Voxel sets consisting of connected nonzero voxels are segmented by considering three‐dimensional 26‐voxel neighborhoods. The volumes of “connected voxel sets” (i.e. the number of voxels enclosed within the set), *V*_*c*_, are subsequently compared with the marker volume, *V*_*Marker*_. Connected voxel sets such that $\frac{1}{2}V_{\mathrm{Marker}}<V_{c}<3V_{\mathrm{Marker}}$ are retained, while all other objects are removed. The reference signal threshold, *R*_*T*_, is defined as the average intensity value of all voxels in connected voxel sets. Connected voxel sets were determined using the standard MATLAB function “bwareaopen.”

#### 2.B.3 Step 3: Size and signal discrimination

Connected voxel sets are assessed for signal intensity and size using an iterative segmentation process using progressively decreasing signal thresholds defined as $S_{T}^{n}={}^{n}/{}_{4}\times R_{T},\text{where}n=7,6,5,4,3,2$ and *R*_*T*_ is the reference signal threshold. With each iteration, threshold $S_{T}^{n}$ is applied to the image and the sets such that 0.35*V*_*Marker*_ < *V*_0_ < 1.3*V*_*Marker*_ are segmented. Results of each iterative step are added to results from the previous step forming a composite three‐dimensional image of the individual marker candidates. If the addition of a new marker candidate yields an object with a volume greater than 1.3*V*_*Marker*_, then that added marker candidate is discarded.

#### 2.B.4 Step 4: Shape discrimination

The marker candidates are evaluated based on their shape. Only those with their longest dimension not exceeding 26.9 mm (1.5 times the known longest dimension of original cylindrical marker) are retained; all other candidates are removed.

#### 2.B.5 Step 5: Inter‐marker distance discrimination

In this last step, the algorithm looks for a group of four marker candidates whose intensity‐weighted centers‐of‐mass are separated by distances equal to the physical inter‐marker separations of the fiducial device. If four candidates are found fitting that condition within the tolerance of ±1 mm, they are consequently identified as the segmented markers of the fiducial device. Otherwise, the separation tolerance is incrementally increased by 1 mm, and the process is repeated until the four markers are found.

### 2.C. Validation experiments

#### 2.C.1. Device location and orientation: Detection rate and repeatability test (Phantom MRI)

MR images can be acquired in arbitrary oblique orientations and with arbitrary table shifts. We used this capability to test robustness of automatic determination of the absolute location and orientation of the fiducial device. The robustness was assessed through the repeatability of both, the determination of the “center‐of‐mass” of the fiducial markers and the device orientation, under varied location and angles of MR acquisition planes. For the purpose of the tests, the fiducial device was placed on a phantom consisting of two cylindrical containers of nickel chloride (Dielectric Corporation, Menomonee Falls, WI), which served as an RF‐load for the coil. The device was placed with its base purely in the coronal plane (i.e. the vector normal to the base of the device was in the vertical direction) with orientation angles of (90$\hat{\vartheta }$, −90$\hat{\phi }$), where $\hat{\vartheta }$ and $\hat{\phi }$ correspond to conventional spherical coordinate angles for a Left‐Posterior‐Superior (LPS) Cartesian coordinate system (patient loaded feet first). Using an 8‐channel receive‐only torso coil, a gradient echo‐based pulse sequence (spoiled gradient‐recalled, SPGR) and a spin echo‐based pulse sequence (fast relaxation fast spin echo, FR‐FSE) were executed to acquire images on a GE Signa HDxt 1.5T MRI (GE Healthcare, Wauwatosa, WI). Selected parameters of these clinical sequences are given in Table 1. The images were collected in three predefined imaging planes: axial (perpendicular to the long axis of the scanner) and two separate oblique planes. The table and the device were then translated 3 cm along the long scanner axis, and similar image sets were acquired. Axial images acquired in the first table position were used as a “baseline” dataset, to which image sets from subsequent acquisitions were compared. For each of the imaging planes, the automated segmentation algorithm was used to detect and segment the markers, as described earlier in the Section 2.B, and subsequently, the position of the fiducial markers’ center‐of‐mass and the device orientation (i.e. the orientation of the vector normal the base) was determined, as shown in Fig. 1. The positions of the translated device obtained using the axial and oblique acquisitions were compared with those of the baseline acquisitions, and the relative displacements were computed. Similarly, the device orientation angles (including those for translated device) determined using oblique acquisitions were then compared with those from the baseline measurements and the angular differences were calculated.

#### 2.C.2. Volunteer trials

The fiducial device was imaged on five volunteers (three males, two females) of different ages and body habitus. Eight sets of images were acquired for each volunteer using the aforementioned MRI scanner, 8‐channel torso coil, and the abdominal pulse sequences from the clinical abdominal protocol listed in Table 1. Following image acquisition, the four markers comprising the fiducial device were individually segmented with our algorithm, and inter‐marker spacings were calculated.

### 2.D. Proof‐of‐concept

**Figure 3:**
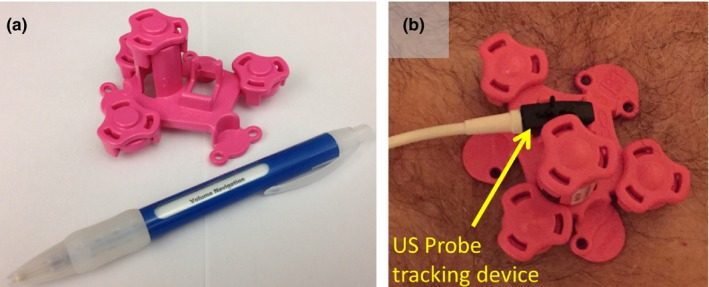
(a) Prototype commercial version of MRI fiducial device used in the phantom and volunteer proof‐of‐concept experiments described in Section 2.D of the Methods. (b) Fiducial device in with a tracking device attached to it.

The research work reported here has led to the development of an integrated, commercial system for automated registration and fusion of MRI data and live US imaging. This system is based on a model LOGIQ E9 ultrasound scanner with Volume Navigation technology (GE Healthcare, Wauwatosa, WI), and the fiducial device shown in Figs. 3(a)–3(b) which was manufactured by CIVCO Medical Solutions (Coralville, IA). Fused MRI and US data from a phantom and human volunteer were obtained with an early version of this system implemented by GE Healthcare as a proof‐of‐concept of the overall feasibility of this automated MRI‐US fusion approach.

MRI was first performed on both an anthropomorphic multimodality abdomen phantom (Model 057A, CIRS: Tissue Simulation and Phantom Technology, Norfolk, Virginia) and a human volunteer with the commercial fiducial device present in the field of view during the scans. Images were acquired on a Discover MR750 scanner (GE Healthcare, Wauwatosa, WI) with a LAVA pulse sequence and using GE‐default acquisition parameters similar to those listed in Table 1. US imaging sessions were performed on the phantom and the volunteer 1 day after the acquisition of MR images. In phantom experiments, the fiducial device was permanently affixed to the phantom for both MR and US imaging. In the volunteer experiments, the position of the fiducial device was marked on the volunteer's skin with ink prior to the MRI scans, and a fiducial device was placed on the volunteer at the marked location for US imaging.

For both the phantom and volunteer experiments, an electromagnetic tracking sensor, which tracks the position and orientation of the ultrasound probe, was affixed to the MRI fiducial device as depicted in Fig. 3(b). This tracking device is one element of the Volume Navigation component that is integrated with the US scanner. For each experiment, the MR image set was loaded onto the US scanner and then processed by the auto‐segmentation algorithm. Following successful segmentation of the device in the 3D MRI dataset, the system automatically identified the plane in the dataset that corresponded with the real‐time ultrasound images. Such image co‐registration is achieved through utilizing the following: (a) The MRI dataset's coordinate system is known relative to the electromagnetic tracking sensor through mechanically coupling (fixed geometry) of the fiducial device and the electromagnetic sensor. (b) The US probe's position and orientation are also known relative to the electromagnetic tracking sensor, the primary attribute of the Volume Navigation feature. (c) US and MRI images are then co‐registered through a mutually shared coordinate system with the electromagnetic tracking sensor. Subsequently, the corresponding MR cross‐sectional images were automatically fused with real‐time US images. Accuracy of the fusion result was evaluated by manually identifying the location of landmarks in both the MR data set and the US images, and then computing the distance in three‐space between corresponding landmark locations.

## 3 RESULTS

### 3.A. Validation experiments

#### 3.A.1. Device location and orientation: Detection rate and repeatability test (Phantom MRI)

All of the markers were accurately segmented in all of the images. The average difference between the actual and image‐derived marker centers‐of‐mass spacings was 0.41 ± 0.37 mm among all images acquired with both SPGR and FR‐FSE pulse sequences. Table 3 shows the center‐of‐mass coordinates calculated based on the baseline acquisition and compares them with those calculated based on acquisitions using oblique scanning planes and translated device, as described in Section 2.C.1. The coordinate differences shown in Table 3 corresponds to average differences in center‐of‐mass locations of 1.4 (SPGR) and 2.1 mm (FR‐FSE) and maximum differences of 1.9 (SPGR) and 2.9 mm (FR‐FSE). Similarly, Table 4 shows orientation angles for the initial, baseline acquisition, and differences in measured orientation angles between the different acquisitions and the baseline acquisition. Angle differences are given for all combinations of MRI sequence, translation, and oblique angulation. The repeatability of the angular measurements is, thus, represented by the maximum absolute angular differences of 0.6° (SPGR) and 0.9° (FR‐FSE).

**Table 3:** Device location in the initial, baseline acquisition, and differences in measured device location between different acquisitions and the baseline acquisition. The device centers‐of‐mass are given in the LPS patient coordinate system and in units of millimeters

Pulse sequence	Dimension	Position (mm)	Position difference (mm)				
		Nontranslated axial	Nontranslated oblique 1	Nontranslated oblique 2	Translated axial	Translated oblique 3	Translated oblique 4
SPGR	*X*	41.73	−0.50	1.09	0.87	−0.10	1.74
	*Y*	−122.88	−0.90	0.88	−0.11	−1.29	0.60
	*Z*	−17.94	−0.85	0.52	−0.09	−0.85	0.48
	Center‐of‐mass	–	1.3	1.5	0.9	1.5	1.9
	Average Δ center‐of‐mass		1.4				
FR‐FSE	*X*	41.43	1.23	−1.78	0.74	−0.75	1.89
	*Y*	−123.05	1.11	−1.70	−0.08	−1.97	1.10
	*Z*	−20.15	−0.25	−1.46	0.37	−1.84	−0.19
	Center‐of‐mass	–	1.7	2.9	0.8	2.8	2.2
	Average Δ center‐of‐mass		2.1				

**Table 4:** Orientation angles for the initial, baseline acquisition, and differences in measured orientation angles between the different acquisitions and the baseline acquisition. The device was placed on a level base, so the anticipated orientation angles were (90$\hat{\vartheta }$–90$\hat{\varphi }$). The direction cosines are listed for each unique acquisition

Pulse sequence	Angle	Angular position (°)	Angular difference (°)				
		Nontranslated axial	Nontranslated oblique 1	Nontranslated oblique 2	Translated axial	Translated oblique 3	Translated oblique 4
SPGR	*Θ*	90.146	−0.030	−0.203	−0.146	−0.557	−0.040
	*Φ*	−87.387	−0.125	0.042	0.146	0.228	0.250
	Direction Cosines	[1;0;0; 0;1;0]	[0.98;0.0004;0.18; −0.038; 0.98; 0.21]	[0.98;0; −0.19; −0.036;0.98; −0.2]	[1;0;0; 0;1;0]	[0.96;0.019;0.27; −0.1;0.96;0.28]	[0.98;0; −0.17; −0.03;0.98;−0.17]
FR‐FSE	*Θ*	90.375	0.491	−0.873	−0.349	0.004	0.427
	*Φ*	−87.690	0.248	−0.165	0.246	0.503	0.144
	Direction Cosines	[1;0;0; 0;1;0]	[0.98;0; −0.12; −0.04;0.98; −0.18]	[0.95; −0.09;0.3; 0;0.95;0.3]	1;0;0; 0;1;0]	[0.96;0;0.3; −0.096;0.94;0.3]	[0.98;0; −0.17; −0.035;0.98;−0.2]

#### 3.A.2. Volunteer trials

**Figure 4:**
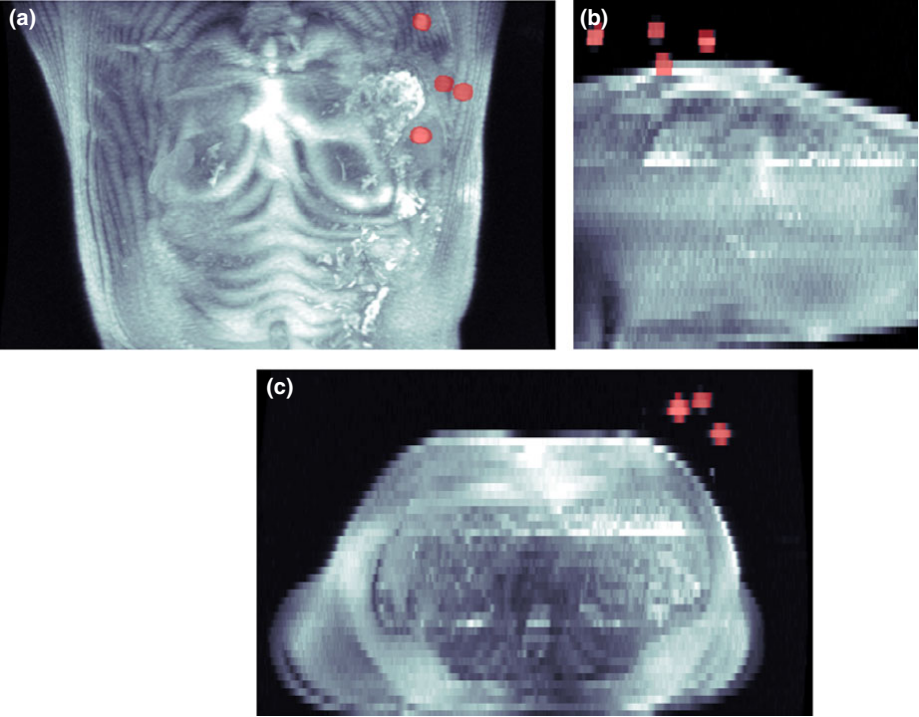
Sample maximum intensity projection (MIP) images from a volunteer dataset acquired with the SS‐FSE pulse sequence. (a) Coronal MIP. (b) Sagittal MIP. (c) Axial MIP. The segmented device is superimposed and shown in red.

Each of the four markers on the device was accurately segmented for all volunteers and image sets. No false positives were recorded. An example set of segmentation results is shown in Fig. 4. To validate the accuracy of the determined marker location and orientation, the algorithm‐based separation measurements were compared with the known, actual inter‐marker separation distances. Average separation distance differences were determined across all volunteers. The global average of absolute separation differences was 0.53 ± 0.36 mm. This value includes all pulse sequences, all marker separation distances, and all volunteers. Separation distances averaged for each unique marker spacing were also examined. In addition, the differences in absolute position of the fiducial device, as compared with the device position in the LAVA image set, were computed. Results are shown in Table 5.

**Table 5:** Actual separation distances between the individual markers that comprise the fiducial device and the average discrepancies between the actual and algorithm‐measured spacings. ΔDevice COM corresponds to the difference in absolute Center‐of‐Mass position of the fiducial device, as compared with the position determined from the LAVA acquisition

Actual spacing (mm)	Separation difference (mm)							
	3 plane loc.	SS‐FSE	IP	OP	LAVA	FIESTA	SPGR	FR‐FSE
88.9	0.6 ± 0.9	−0.4 ± 1	0.2 ± 0.9	−0.5 ± 0.7	0.2 ± 0.8	0.1 ± 0.5	0.4 ± 0.9	1.6 ± 3.7
69.2	1.5 ± 2	−0.8 ± 1	0.1 ± 0.6	−0.6 ± 2	0.3 ± 0.9	0.3 ± 0.7	0.4 ± 0.9	0.4 ± 0.9
51.9	0.7 ± 1.5	0.2 ± 0.7	0.6 ± 1.1	−0.3 ± 0.8	0.9 ± 1	0.7 ± 0.4	1.2 ± 1.7	0.5 ± 0.8
50.7	−1 ± 1.7	0.4 ± 0.4	−0.1 ± 1.2	0.1 ± 0.5	−0.1 ± 0.5	0.6 ± 1	0.2 ± 0.8	−0.4 ± 1
44.3	0.9 ± 1.7	0.5 ± 1	−0.4 ± 0.7	0.3 ± 0.7	−0.7 ± 1	−0.3 ± 0.8	−0.8 ± 1.2	0.3 ± 2.7
30.3	1.1 ± 1.2	0.3 ± 0.5	0.1 ± 1.2	−0.4 ± 2.4	0.4 ± 0.3	0.8 ± 1.2	0.5 ± 0.6	1 ± 1.1
Δ Device COM	4.8 ± 1.5	10.7 ± 1.8	1.7 ± 0.7	1.7 ± 0.7	NA	1.0 ± 0.2	1.7 ± 0.8	6.7 ± 6.2

### 3.C. Proof‐of‐concept

**Figure 5:**
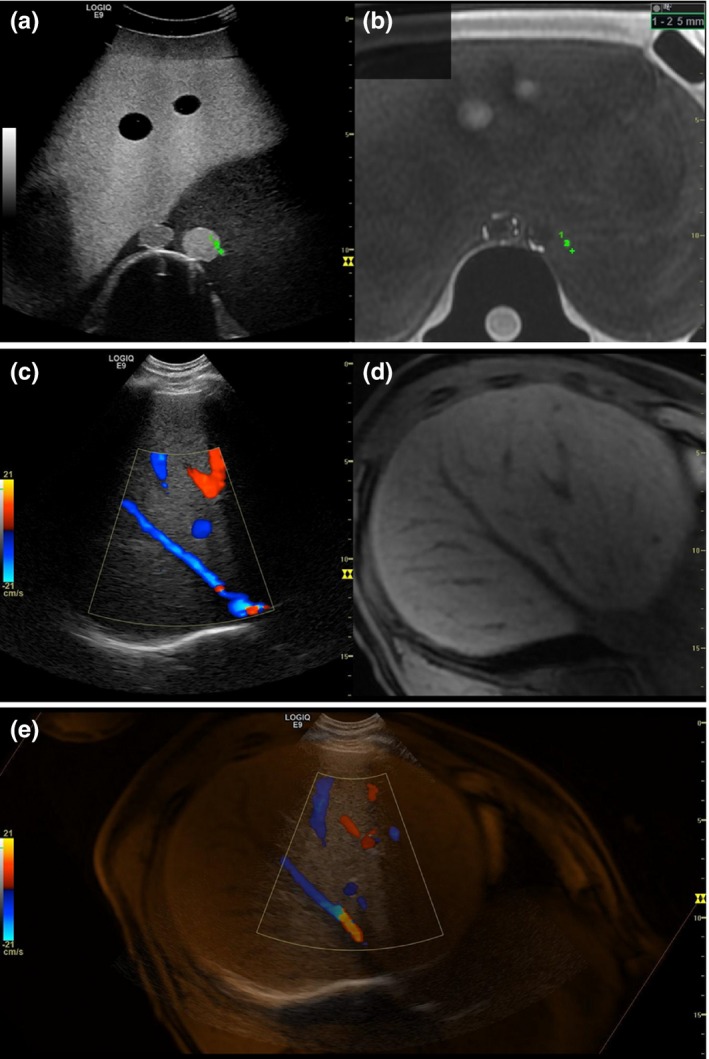
Fused US (a) and MR (b) images of the multimodality phantom. The green points in (a) and (b) indicate the shared landmark locations used to measure image fusion accuracy. Fused US color Doppler (c) and MR (d) images of the volunteer's abdomen. (e) Overlay of the fused MR and color Doppler US images acquired in the abdomen of the volunteer.

Using the integrated, commercial system, the fiducial device was located with the auto‐segmentation algorithm in both the phantom and volunteer experiments. MR‐US fused images were immediately displayed following auto‐registration. Example screen capture images are shown in Fig. 5. In the fused phantom images, user‐identified landmarks visible in both US and MR images were measured to be 5 mm apart, in‐plane. In the volunteer experiment, the landmark chosen to assess image fusion accuracy was the bifurcation of the left portal vein. The location of this bifurcation during US imaging was determined both while the volunteered executed a breath‐hold (as in the MRI acquisition) and while breathing freely. In‐plane separation measurements yielded discrepancies between landmark locations of 5 and 13 mm, with and without a breath‐hold, respectively. The software then allowed further, optional user input to manually align and match locations of the landmark on both MR and US images.

## 4 DISCUSSION

In every experiment, all four fiducial markers that comprise the device were automatically detected in 100% of the trials, including all phantom and volunteer images, pulse sequences, and device orientations. The algorithm accurately determined the device center‐of‐mass and orientation across different acquisition angles. The inter‐marker spacings, as measured with the algorithm, were accurate for both the phantom and volunteer experiments. Most separation distance measurements were accurate to within less than 1 mm of the actual inter‐marker separation distances, which corresponds to the maximum 1 mm engineering tolerance between any two markers in the physical device.

In addition, segmentation was robust in the volunteer experiments across all pulse sequences, including the 3‐plane localizer, in which 10 mm thick image slices were generated; no pulse sequence demonstrated significantly different separation measurements. One of the greatest hurdles in detection is to ensure that each marker signal is generated and collected in the image. Abdominal imaging employs some of the largest slice thicknesses and slice gaps of all MRI applications. These results are suggestive of wide applicability of this device and algorithm for use in areas outside of abdominal imaging.

The proof‐of‐concept experiments demonstrated the successful application of this automated fusion approach. Fused breath‐hold volunteer images were accurate within 5 mm, measured as the difference between user‐defined landmark locations visible in both MR and US images. This accuracy was better than variations resulting from respiratory motion (approximately 8 mm). With this particular implementation of the automated fusion system, a “first‐order” co‐registration is simple and efficient, requiring only a few steps, and is completed in under half a minute. For some clinical applications, image fusion accuracy within 5 mm may be insufficient; however, if improved, local accuracy is needed, the user has the option to manually refine the co‐registration. In addition, co‐registration error may propagate and become too large for deep lesions. This optional function would enable the user to mitigate this scenario. In our current clinical US‐guided ablation practice, registration is very often done in two manual steps, one to establish a global match between modalities and the second to optimize local registration for each lesion to be treated. We believe making the initial global registration step automatic, as our approach is designed to do, would offer a significant advantage in these procedures.

There are several minor limitations associated with the application of this device. To capture all of the external markers in the image, it may be necessary to expand the MR imaging field of view, which poses an additional task for the technologist and could result in a slightly longer scan time. The first prototype device was investigated using an 8‐channel phased‐array torso coil from a single vendor, which has large open regions in the center of the surface coils to place the device. Although we have observed similar sized open regions in surface coils from other vendors, some coil arrays may not work as well with this device in its current size. In such a scenario, the device could be modified to fit the physical demands of the specific set of coils or simply placed underneath the coil element(s). Additional issues regarding patient respiratory and organ motion along with tissue deformation from the US probe are not addressed by this automated fusion system and will continue to cause potential complications for MR‐US fusion applications.

**Figure 6:**
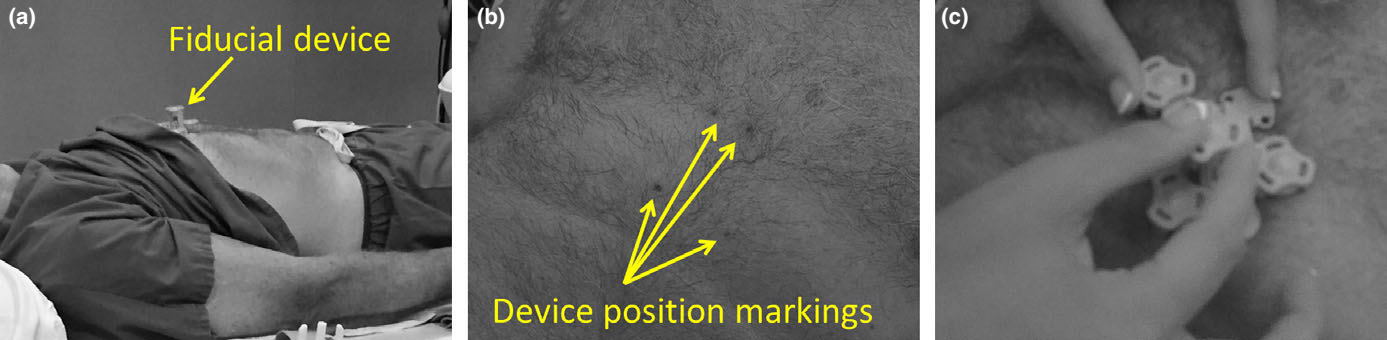
(a) Photo of the prototype fiducial device place on the volunteer. (b) Photo of the ink markings indicating the device position during MRI. (c) Photo of the replacement of the device for US imaging.

From a practical standpoint, some workflow complications would have to be addressed. The MRI fiducial device needs to be placed on the patient for their MRI examination. Thus, patients who require MR‐US fusion must be identified prior to their MRI examination or the examination must be fully or partially repeated with the device in place. This may be challenging in clinical practice. The current system enables ink markings of the fiducial device's positon for accurate re‐position of the device (Fig. 6); however, additional investigations on device replacement repeatability, placement location on the patient, and general workflow—specific to a given clinical practice—would greatly aid the successful implementation of this system.

Although tests of this system were successful, the following future developments of this system could improve its robustness and clinical applicability. The algorithm can be modified to find the center‐of‐mass of the device if only three markers are successfully segmented. Moreover, an option for user input to identify the location of the markers would mitigate a complete failure to segment the markers. In addition, different device shapes and marker sizes could be tailored for specific applications. The device in this investigation was designed with abdominal applications in mind. For instance, smaller markers (and a smaller device footprint) could be used for MRI examinations that reconstruct thinner slices with thinner slice gaps. Also, all image acquisitions from the entire examination could be determined and averaged with appropriate (resolution‐based) weighting factors applied for each sequence, which could possibly provide more accurate localization and further reduce the likelihood of localization failure. Lastly, this system could be combined with deformable image registration techniques that require an initial three‐point registration of the images,^14^ in which case this system would provide the necessary initial global rigid registration.

## 5 CONCLUSION

The work presented in this report demonstrates that the two‐component MRI fiducial marker system (passive fiducial device coupled with the detection algorithm) enables robust, accurate, and reliable detection and localization of the fiducial device within MR image sets. Moreover, the proof‐of‐concept experiments demonstrate that implementation of this automatic MR‐US image fusion approach on clinical US scanners is possible and results in accurately fused images readily displayed on an ultrasound scanner display.

## CONFLICT OF INTEREST

M. Washburn: Employee; GE Healthcare.
